# Application of the 3D slicer chest imaging platform segmentation algorithm for large lung nodule delineation

**DOI:** 10.1371/journal.pone.0178944

**Published:** 2017-06-08

**Authors:** Stephen S. F. Yip, Chintan Parmar, Daniel Blezek, Raul San Jose Estepar, Steve Pieper, John Kim, Hugo J. W. L. Aerts

**Affiliations:** 1 Department of Radiation Oncology, Dana-Farber Cancer Institute, Brigham and Women’s Hospital, and Harvard Medical School, Boston, MA, United States of America; 2 Biomedical Engineering Department, Mayo Graduate School of Medicine Rochester, MN, United States of America; 3 Department of Radiology, Brigham and Women’s Hospital and Harvard Medical School, Boston, MA, United States of America; 4 Isomics, Inc., Cambridge, MA, United States of America; 5 Department of Radiology, University of Michigan Health System, Ann Arbor MI, United States of America; University of Groningen, University Medical Center Groningen, NETHERLANDS

## Abstract

**Purpose:**

Accurate segmentation of lung nodules is crucial in the development of imaging biomarkers for predicting malignancy of the nodules. Manual segmentation is time consuming and affected by inter-observer variability. We evaluated the robustness and accuracy of a publically available semiautomatic segmentation algorithm that is implemented in the 3D Slicer Chest Imaging Platform (CIP) and compared it with the performance of manual segmentation.

**Methods:**

CT images of 354 manually segmented nodules were downloaded from the LIDC database. Four radiologists performed the manual segmentation and assessed various nodule characteristics. The semiautomatic CIP segmentation was initialized using the centroid of the manual segmentations, thereby generating four contours for each nodule. The robustness of both segmentation methods was assessed using the region of uncertainty (δ) and Dice similarity index (DSI). The robustness of the segmentation methods was compared using the Wilcoxon-signed rank test (p_Wilcoxon_<0.05). The Dice similarity index (DSI_Agree_) between the manual and CIP segmentations was computed to estimate the accuracy of the semiautomatic contours.

**Results:**

The median computational time of the CIP segmentation was 10 s. The median CIP and manually segmented volumes were 477 ml and 309 ml, respectively. CIP segmentations were significantly more robust than manual segmentations (median δ_CIP_ = 14ml, median dsi_CIP_ = 99% vs. median δ_manual_ = 222ml, median dsi_manual_ = 82%) with p_Wilcoxon_~10^−16^. The agreement between CIP and manual segmentations had a median DSI_Agree_ of 60%. While 13% (47/354) of the nodules did not require any manual adjustment, minor to substantial manual adjustments were needed for 87% (305/354) of the nodules. CIP segmentations were observed to perform poorly (median DSI_Agree_≈50%) for non-/sub-solid nodules with subtle appearances and poorly defined boundaries.

**Conclusion:**

Semi-automatic CIP segmentation can potentially reduce the physician workload for 13% of nodules owing to its computational efficiency and superior stability compared to manual segmentation. Although manual adjustment is needed for many cases, CIP segmentation provides a preliminary contour for physicians as a starting point.

## Introduction

Quantitative imaging has become an important area of research for the development of non-invasive imaging biomarkers for numerous applications, such as the prediction of clinical outcomes, and assessment of treatment response and gene expression [1, 2]. In particular, quantitative imaging has the potential to have an immense impact on lung cancer patients. Lung cancer is a leading cause of cancer-related death among men and women, affecting over 1.8 million patients worldwide [3]. At the time of diagnosis, the majority of patients are in advanced stages of disease, resulting in poor prognoses with a 5-year overall survival rate of < 20% [4]. However, patients who are treated for early stage disease have a substantially greater overall survival rate of > 50% [4]. Therefore, identification of patients with early stage disease is crucial for improving prognosis of lung cancer patients [5].

Computed tomography (CT) is routinely used to diagnose and monitor disease progression in lung cancer patients, where early stage disease is often manifested as pulmonary nodules [6, 7]. One of the challenges of identifying patients with early stage lung cancer is that these pulmonary nodules may also be an indicator of other benign conditions, such as inflammation and/or infection, rather than malignancy [8]. Studies have hypothesized that malignant nodules possess distinctive CT imaging features from benign nodules, such as greater lesion volume, longer diameter and faster growth rate [9–14]. Classifiers that are built using imaging features have shown promise in assisting physicians to effectively identify different nodule types [15–20]. The development and accuracy of these classifiers relies on accurate delineation of the region of interest that conforms only to the nodule boundaries. Quantitative imaging features are then extracted and evaluated from this region of interest to generate the classifier. Therefore, inaccurate segmentation of tumors can lead to the development of inaccurate classifiers or biomarkers. Manual segmentation by experienced radiologists is commonly used for defining the nodule volume (or region of interest) using a slice-by-slice approach. However, manual segmentation is not only labor intensive, but is also impacted by inter- and intra-observer variability [21–24]. A number of automatic and semiautomatic segmentation methods have been proposed, ranging from simple approaches, such as thresholding [25] and region growing [26], to more complex methods based on the probability map of nodule textures and convexity [17, 27, 28]. Despite having great potential to reduce human errors and expedite the nodule contouring workflow, these methods are currently not publically accessible, which limits their widespread use in clinical and biomedical research.

Alternatively, 3D Slicer is an open-source software platform for biomedical research [29] that supports versatile visualization and provides advanced analysis tools, such as image segmentation and registration. An algorithm implemented in 3D Slicer, known as GrowCut, can delineate large lung tumor volumes more robustly than manual segmentation [30], and reliably extract imaging features for the development of imaging biomarkers [31]. However, segmentation of pulmonary nodules presents a unique challenge since the nodules are often smaller and in close proximity with surrounding tissues. Therefore, additional pruning steps are required in the nodule segmentation process to remove pleural and/or vessel attachments [32, 33]. To address these challenges with nodule segmentation, a level set-based algorithm has been implemented within the Chest Imaging Platform (CIP) in 3D Slicer [33, 34]. This algorithm is based on a front propagation approach from a “seed point” placed within the nodule. The propagation of the front (or segmentation) is constrained to prevent leakage into the chest wall, airway walls or regions with appearance of tubular or vessel-like structures.

This study investigated the ability of CIP segmentation to assist physicians with nodule segmentation. In particular, we evaluated the robustness of the CIP segmentation algorithm in delineating lung nodules and compared its performance with the manual segmentations. The accuracy of the CIP segmentation algorithm and nodule characteristics that could affect the segmentation quality was also investigated.

## Materials and methods

Patient dataset: Since a publicly available dataset was used in this study, approval by an institutional review board was not needed. A publicly available thoracic CT dataset, known as the Lung Image Database Consortium (LIDC), was downloaded from The Cancer Imaging Archive (TCIA: https://wiki.cancerimagingarchive.net/display/Public/LIDC-IDRI/) [23]. The LIDC dataset consisted of 1007 patients with low dose helical thoracic CT images containing annotated lung nodules that were acquired from seven academic institutions with slice thicknesses ranging from 1 mm to 5 mm. In the LIDC dataset, each nodule had 1 to 4 manual segmentations that were performed on a slice-by-slice basis by experienced thoracic radiologists. The LIDC radiologists assigned scores (ranging from 1 to 5) to each nodule for nine categories that described the nodule characteristics, including its subtlety, internal structure, roundness, margin sharpness, lobulation, spiculation, texture, and likelihood of being malignant. Table 1 contains an annotation of the scoring system. Images were excluded from the current study if they were not segmented by 4 or more radiologists (n = 596), nodule numbers were mislabeled (n = 60) or had imaging artifacts (n = 77, Fig A in S1 File). The imaging artifacts were due to corruption in the original LIDC DICOM files. As a result, images from 274 patients with 354 nodules (1–4 nodules/patient) were used to analyze the robustness and accuracy of 3D Slicer CIP-segmentation.

**Table 1:** Annotation of nodule characteristic scoring.

	1	2	3	4	5	6
Nodule Characteristics						
Subtlety	Extremely Subtle	Moderately Subtle	Fairly Subtle	Moderately Obvious	Obvious	N/A
Internal Structure	Soft Tissue (default)	Fluid	Fat	Air	N/A	N/A
Calcification	Popcorn	Laminated	Solid	Non-Central	Central	Absent
Sphericity (Roundness)	Linear		Ovoid		Round	
Margin	Poorly				Sharp	N/A
Lobulation	None				Marked	N/A
Spiculation	None				Marked	N/A
Texture	Non-Solid/Ground Glass Opacity		Part Solid/Mixed		Solid	N/A
Malignancy	Highly unlikely	Moderately unlikely	Indeterminate	Moderately suspicious	Highly suspicious	N/A

The intermediate values for Roundness, Margin, Lobulation, Spiculation, and Texture are allowed to use by the radiologists. N/A = not applicable.

Nodule segmentation algorithm in 3D Slicer: The CIP in 3D Slicer 4.5 [29] employs semiautomatic nodule segmentation algorithm based on the open-source a Lesion Sizing Toolkit-based [33]. A seed point is placed within the nodule region to initialize the segmentation. In this current study, the seed point was chosen as the centroid of each manual contour. As there were four radiologist-defined nodule contours, four CIP-segmentations were generated automatically for each nodule. To speed up the computation time, the CIP segmentation algorithm also automatically cropped the CT images around the seed points with a radius of 30 mm. If an image consisted of multiple nodules, then an automatically cropped region was created around the seed point for each nodule.

Within each cropped region, the nodule segmentation was based on a level set formulation (Sethian 1999) to propagate a front according to a Geodesic Active Contour functional [35]. The contour propagation is governed by a smoothing term that minimizes the curvature of the contour and an “attachment” term that pulls the front towards the features of interest. This second term employs a speed map, F, to guide the segmentation results according to the desired characteristics of nodules. The speed map is obtained as a sigmoid transformed min pooling of four different feature maps that are designed to slow down the evolution in 1) the chest wall region, 2) vascular structures, 3) the interface between the nodule and the lung parenchyma, and 4) areas whose density is not compatible with nodular structures. The chest wall feature map was obtained according to a threshold-based approach followed by morphological operations similar to the ones employed in standard lung segmentation approaches [36]. Vessel-like structures were penalized based on the Sato vesselness filter [37]. The interface map between the nodule and the lung parenchyma is defined according to a canny edge detector. Finally, the non-nodular regions were excluded based on a sigmoid function with parameters, alpha = 100 and beta = -200 and -500 for solid and non-solid nodules respectively. One or more seed points within the nodule initialize the segmentation.

**Fig 1:**
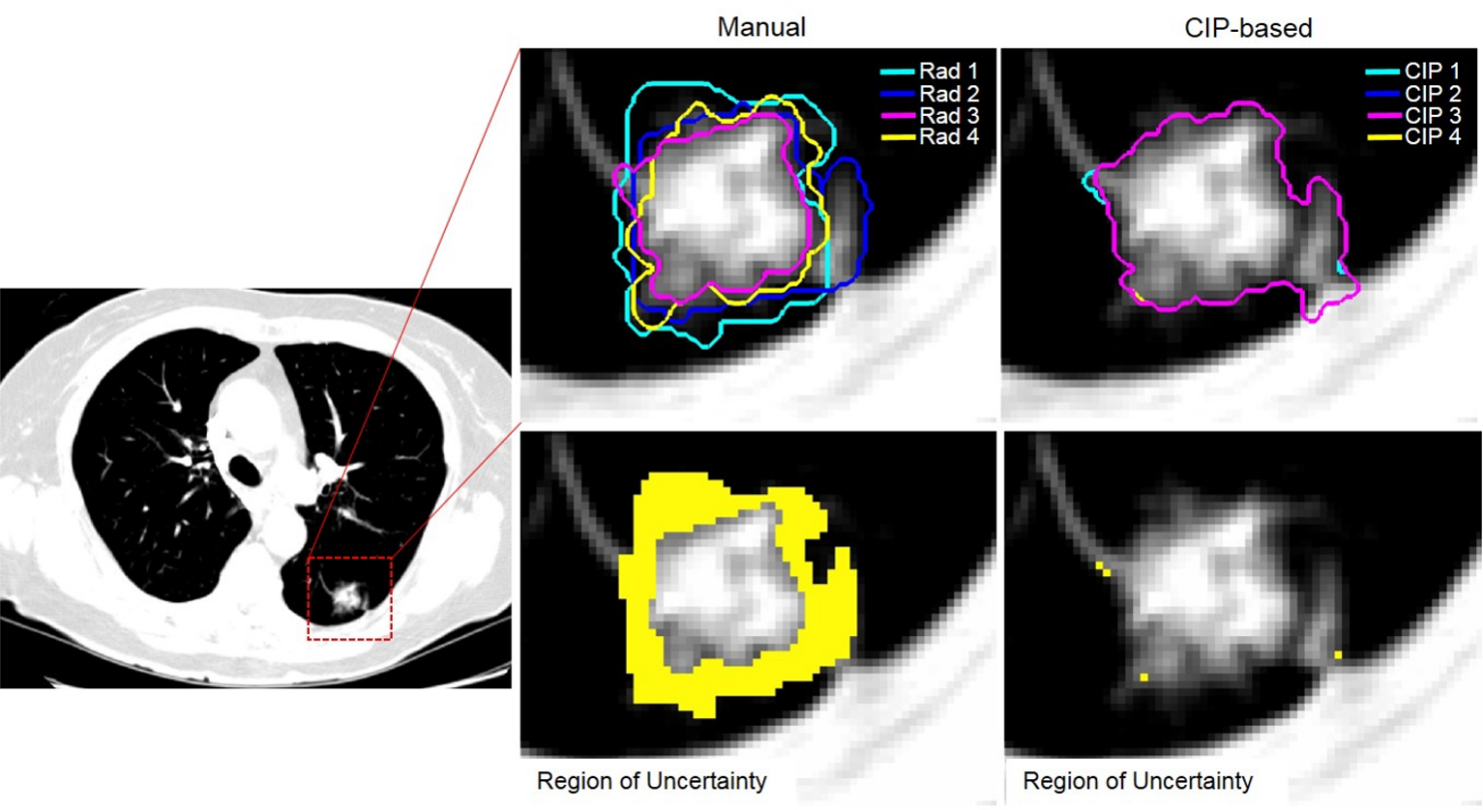
Comparison of manual (left) and CIP-based (right) segmentation. Yellow shaded region indicated the disagreement (or region of uncertainty) between contours performed by four radiologists (bottom left) or different CIP-based seed locations (bottom right). In this example, the region of uncertainty for manual segmentation was 3222 ml while the region was only 46 ml for the CIP-based segmentation. dsi_CIP_ was ≈ 100%, while dsi_manual_ was 88%.

Robustness of the segmentation methods: The region of uncertainty (δ) and dice similarity index (DSI), were used to assess the robustness of the manual and CIP segmentations. The region of uncertainty was defined as the negation of the intersect regions of all the segmentations (Fig 1). In particular, the region of uncertainty (δ) was defined as follow:

$$\delta_{method}=(V_{method}^{I}\cup V_{method}^{II}\cup V_{method}^{III}\cup V_{method}^{IV})-(V_{method}^{I}\cap V_{method}^{II}\cap V_{method}^{III}\cap V_{method}^{IV})$$ (1)

**Fig 2:**
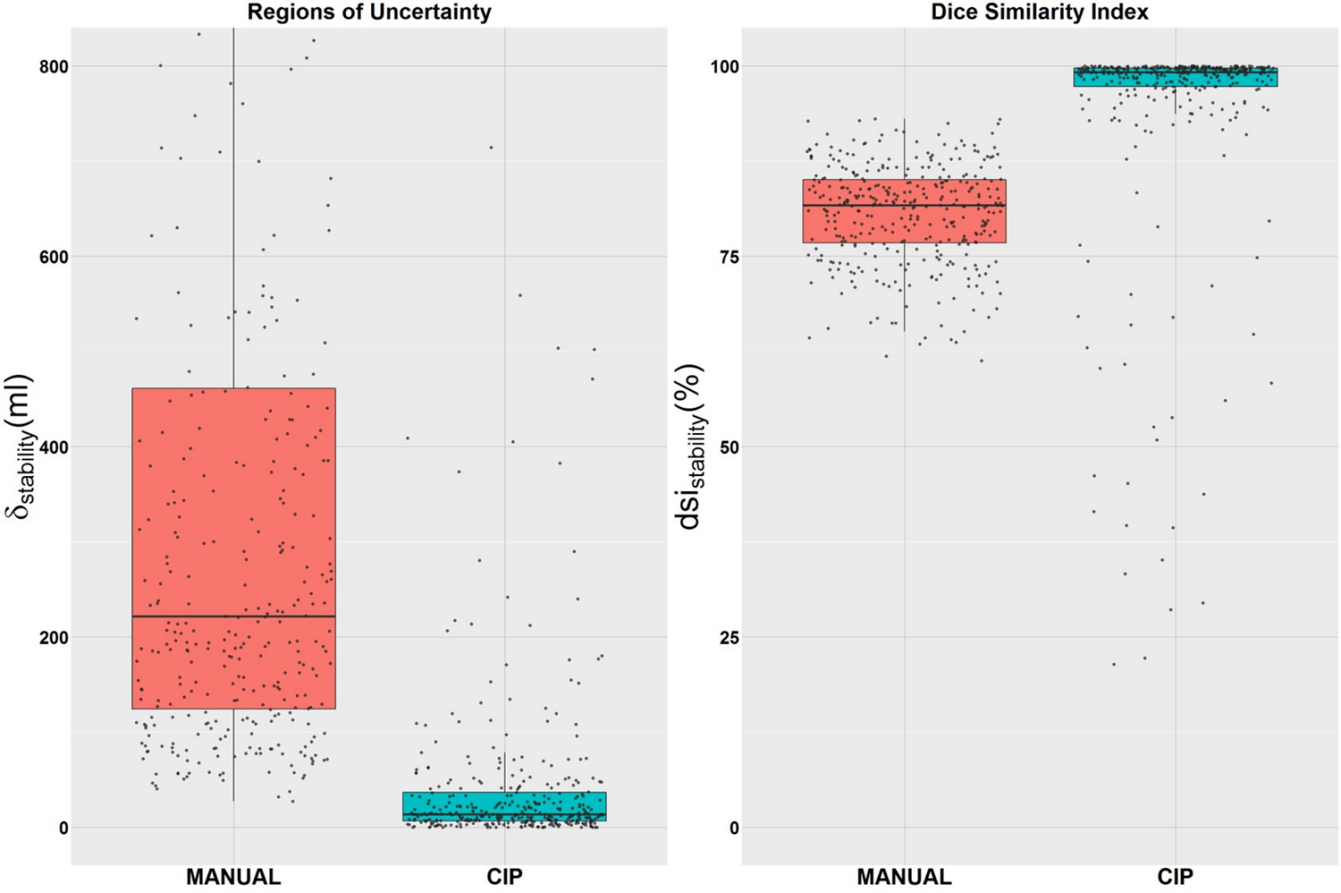
Robustness (or stability) of the manual and CIP-based segmentation. The robustness of the manual and CIP-based segmentation assessed with the region of uncertainty (δ) and Dice similarity index (dsi).

Where *method* could either be manual or CIP segmentation. For manual segmentation, the superscript indicates the nodule volume delineated by the four different radiologists, whereas for CIP segmentation, it indicates the segmentations initialized by the centroid computed from the four radiologist-defined volumes. δ equaled to zero indicated that the segmentation method was perfectly robust across the four segmentations. The stability of the segmentation method decreases with increasing in the δ_method_ (Fig 2).

The DSI for segmentation stability was defined as follow:

$${dsi}_{method}=\frac{1}{6}\sum_{i\neq j}\frac{{n(V}_{method}^{i}⋂V_{method}^{j})}{\frac{{n(V}_{method}^{i})+n(V_{method}^{j})}{2}}∙100\%$$ (2)

Where *method* could either be manual or CIP segmentation. n(V) indicates the number of voxel in volume V. *i* and *j* ranged from 1 to 4 indicating nodule volumes segmented by radiologist *i* and *j*, or initialized by the centroid computed from radiologist *i* and *j*, for manual and CIP segmentations, respectively. There were four contours for each segmentation method and, thus, six possible combinations of *i* and *j*. The stability of the segmentation method increases with increasing dsi_method_, where dsi_method_ = 100% indicates a perfectly robust method.

The robustness of the CIP segmentation method (δ_CIP_ or dsi_CIP_) was compared with the manual segmentation method (δ_manual_ or dsi_manual_) using the Wilcoxon signed-rank test, where p_Wilcoxon_ < 0.05 indicated statistical significance. Moreover, the average nodule volume segmented by the manual and CIP contouring methods were also compared and tested for significant differences. The average nodule volume was defined as $\bar{V_{method}}=\frac{1}{4}\sum_{i=1}^{4}V_{method}^{i}$; where *i* indicates radiologist *i*. Since the ground truth of the nodule segmentation is unknown, the average nodule volume ($\bar{V_{manual}}$) computed from the manual contours was used to estimate the true nodule volume. Unless otherwise specified, $\bar{V_{manual}}$ is referred to as nodule volume.

**Fig 3:**
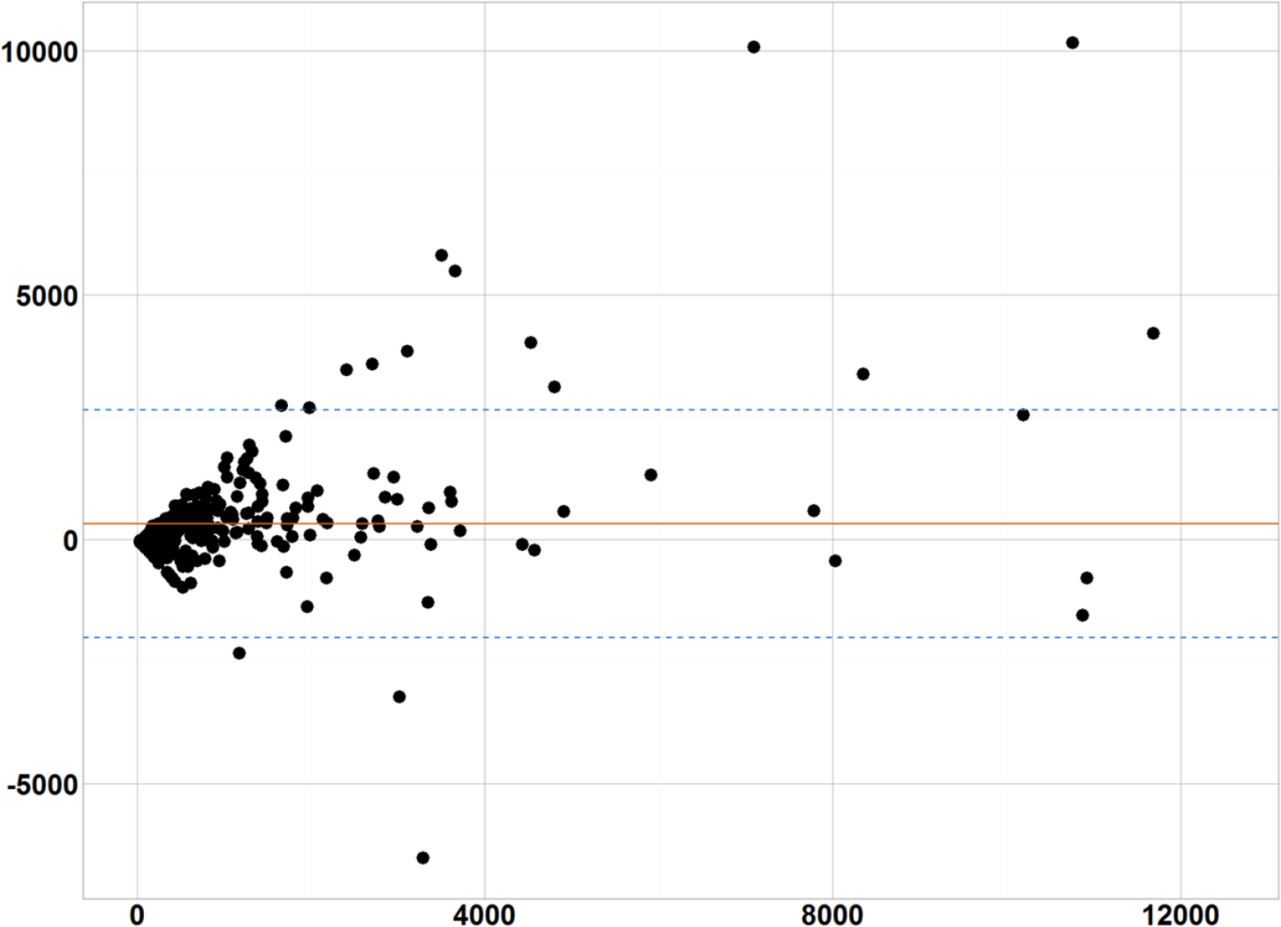
Bland-Altman plots. Bland-Altman plots highlights the differences between $\bar{V_{CIP}}$ and $\bar{V_{manual}}$ for all nodules. The 95% interval of the differences are depicted by the blue dotted lines. Solid red line is the average difference between $\bar{V_{CIP}}$ and $\bar{V_{manual}}$ (= 318ml).

**Fig 4:**
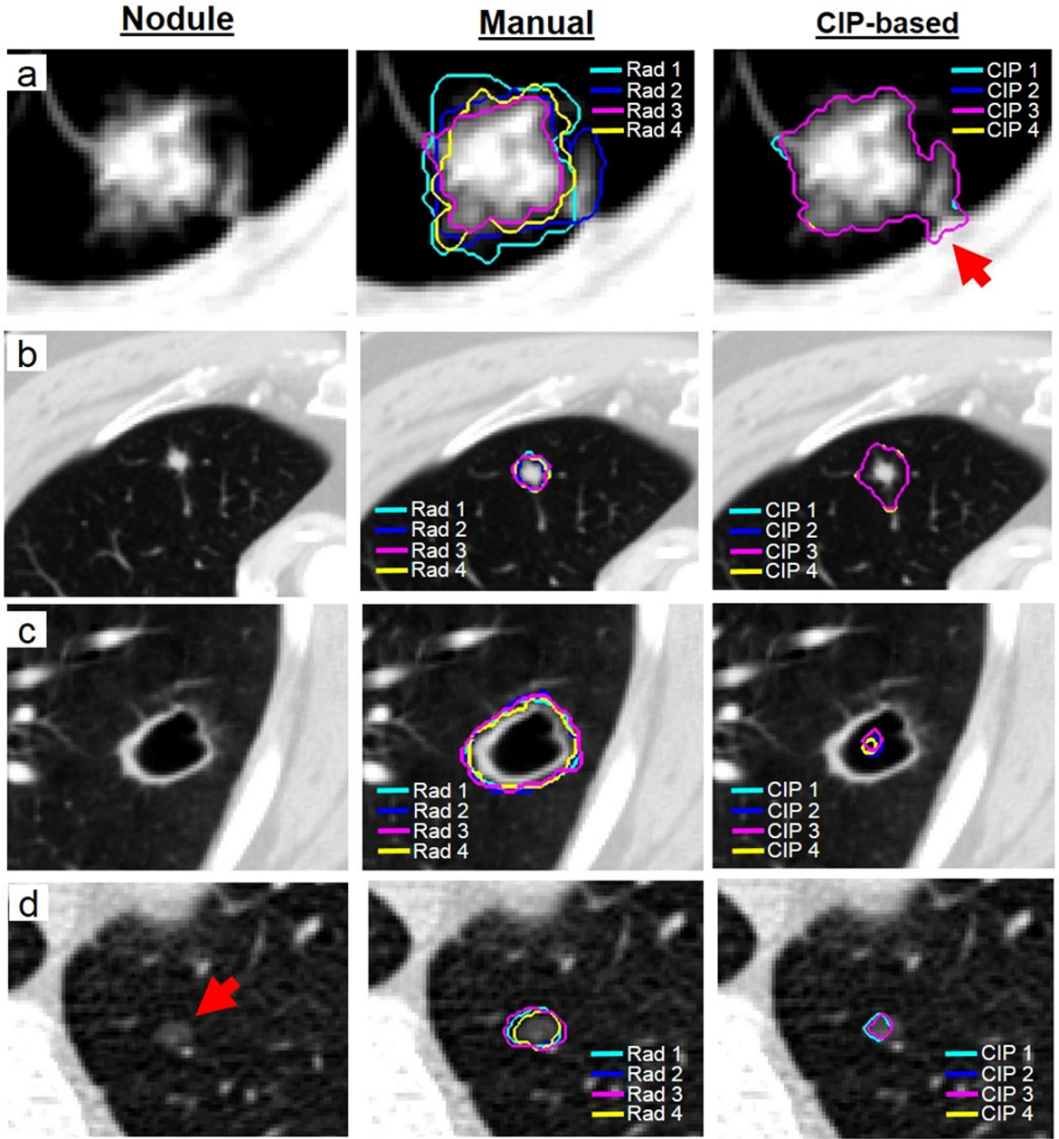
Examples of nodules that were segmented by radiologists manually and CIP segmentations. a) The robustness of the CIP segmentation was excellent, while substantial interobserver variability was observed in manual segmentation. CIP segmentation was also in excellent agreement with manual contours. However, CIP segmentation was observed to include part of the chest wall (indicated by an arrow) b) Despite being perfectly robust CIP segmentation, it included the region of the normal lung in proximity of the small nodule. c) Cavitation in the center of the nodule. Poor CIP segmentation performance was found. d) Non-solid (ground glass opacity) nodule with poorly defined boundary and subtle appearance is indicated by the red arrow. Poor CIP segmentation performance was found.

Accuracy of the CIP segmentation method: The accuracy of CIP segmentations was evaluated to ensure that non-nodular tissues were excluded and the entire nodule volume was contoured. Even if the CIP segmentation was perfectly robust, it may include nearby non-nodule tissues or fail to capture the entire nodule region. For example, despite being almost perfectly robust, nodules contoured by the CIP segmentation method were observed to include substantial normal lung regions as shown in Figs 3B and 4A. The agreement between the manual and CIP segmentations was used to estimate how well the nodule volume could be delineated by the CIP segmentation. DSI_Agree_ was used to assess for the segmentation agreement and was defined as follow:

$${DSI}_{Agree}=\frac{1}{4}\sum_{i=1}^{4}\frac{n(V_{CIP}^{i}⋂V_{j})}{\frac{{n(V}_{CIP}^{i})+n(V_{j})}{2}}∙100\%$$ (3)

Where $V_{CIP}^{i}$ is the CIP segmentation nodule volume initialized by the centroid of nodule volume segmented by radiologist *i*. *V*_*j*_ could either be the intersection (j = 1) or the union (j = 1) of the radiologist defined segmentations.

To avoid confusion, lower case dsi was used to indicate the robustness of the segmentation method while upper case DSI was used to indicate the accuracy of the CIP-based segmentation in this paper.

Moreover, all the CIP-segmentations were visually inspected by an experienced radiologist (J.K.) and researcher (S.Y.). They then classified the nodule segmentations into four categories: 1) substantial, 2) moderate, 3) minor, and 4) no manual adjustment required.

Relationship between nodule characteristics and CIP segmentation accuracy: To identify nodule characteristics that may affect the accuracy of the CIP segmentation, the Spearman’s correlation coefficient was computed between the radiologists scored nodule characteristics and DSI_Agree_. For nodule characteristics that had a continuous scoring scale (e.g. margin ranges from 1 to 5, where 1 indicates a poorly defined margin and 5 indicates a sharp margin) (Table 1), a t-test was used to assess if the correlation coefficient was significantly different from 0 (p_t-test_<0.05). For characteristic categories where the scoring scale was categorical (ordinal) rather than continuous (i.e. nodule calcification where each score indicates a different appearance) (Table 1), the Kruskal-Wallis test (p_Kruskal-Wallis_<0.05) was used.

The correlations between $\bar{V_{manual}}$, DSI_Agree_ and all nodule characteristics were also calculated. Four radiologists scored each category, and thus, there was some variability in the characteristic scoring. When there was a heterogeneous rating, the score that was assigned by the majority of radiologists was chosen for the analysis. In the case of a tie rating, the score that were most frequently assigned to the patient population was chosen. The distributions of the scores for each nodule characteristic are shown in Fig B in S1 File.

Furthermore, Spearman’s correlation coefficient was employed between image voxel thickness and DSI_Agree_ to investigate if the voxel thickness affected the segmentation quality. The significance of the relationship was assessed by a t-test (p_t-test_<0.05).

## Results

In this study, a semiautomatic segmentation method implemented in the CIP of 3D Slicer was used to contour 354 nodules. The computation time of the CIP segmentations was 5–79 s (median: 10s) on a personal computer with 16GB RAM and 3.40GHz Core i7-4770 CPU.

Robustness of the segmentation methods: For the CIP segmentation method, the median dsi_CIP_ was 99% (Interquartile (IQR) range: 97–100%) and the median δ_CIP_ was 14 ml (IQR range: 7–37 ml), while for the manual segmentation method, dsi_manual_ was 82% (IQR range: 77–85%) and the median δ_manual_ was 222 ml (IQR range: 124–461 ml) (Fig 2). Although both segmentation methods were generally robust (median dsi>80%), CIP segmentations were significantly more stable than the manual segmentations with p_Wilcoxon_~10^−16^ for both robustness measures. Fig 4A shows a visual example of a patient with more stable nodule contours by the CIP segmentation method than by the manual segmentation method.

Accuracy of the CIP segmentation: The Bland-Altman plot in Fig 3 highlights the differences between $\bar{V_{manual}}$ and $\bar{V_{CIP}}$ for all nodules. The median value of $\bar{V_{manual}}$ was 309ml (IQR range: 162–796ml) and $\bar{V_{CIP}}$ was 477ml (IQR range: 153–1290ml). Nodules segmented by the CIP method were significantly greater in volume than those by manual method (p_Wilcoxon_~10^−12^). Fig 4B shows an example where CIP segmentation overestimated the nodule region, including parts of the normal lung.

**Fig 5:**
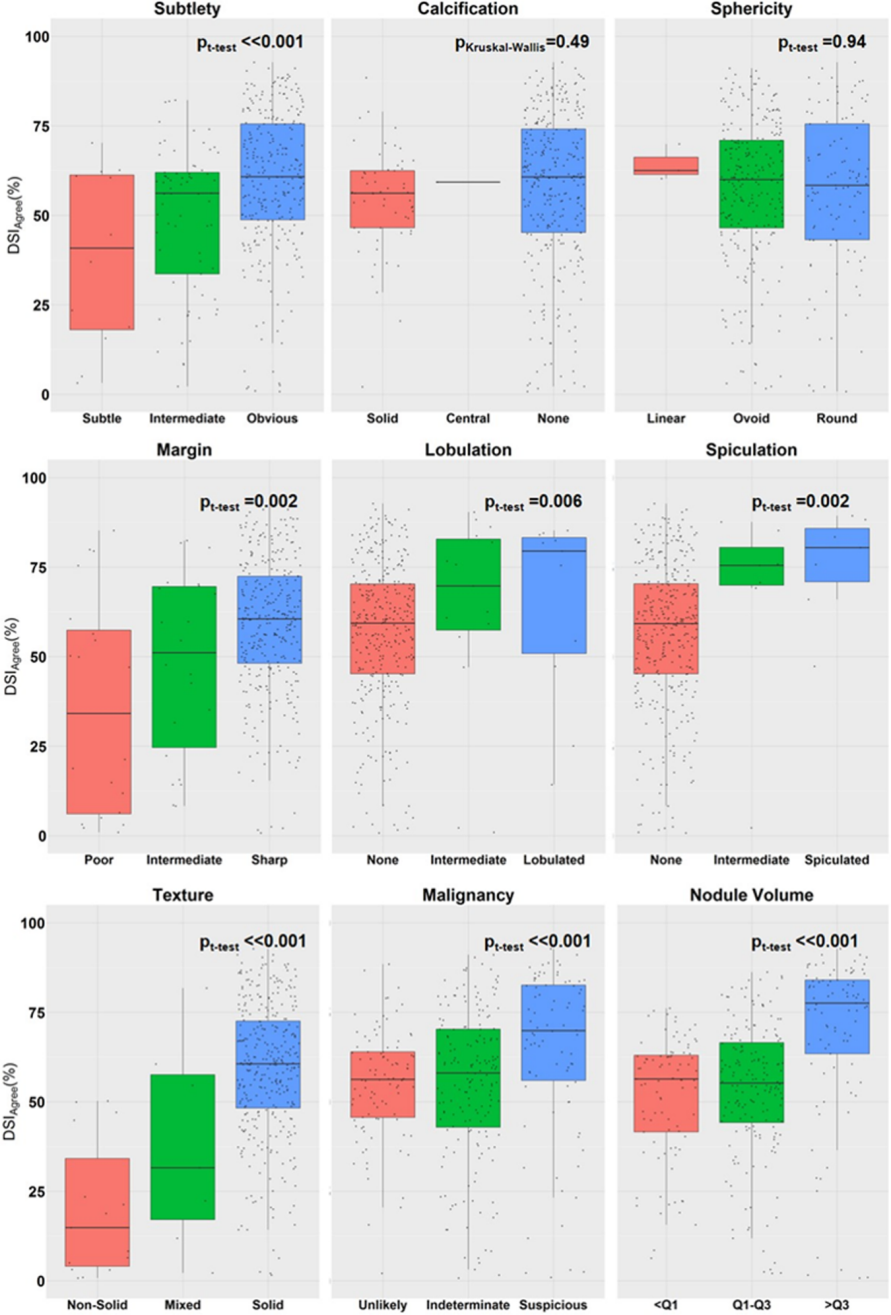
The relationships between nodule characteristics, nodule volume, and DSI_Agree_. This figure highlights the relationships between nodule characteristics, nodule volume, and DSI_Agree_. Calcification: Solid = solid calcification, Central = central calcification, None = no calcification. Lobulation: None = not lobulated. Spiculated: None = not spiculated. Texture: Mixed = Semi-solid nodules. Malignancy: Unlikely = unlikely for cancer, Suspicious = suspicious for cancer. Nodule Volume: Q1 = 162ml, Q1–Q3 = 162ml to 796ml, and Q3 = 796ml; Q = quantile.

The agreement between CIP and manual segmentations that was assessed by the median DSI was 60% (IQR range: 46–71%). The relationship between various nodules characteristics and the accuracy of the CIP segmentation (i.e. DSI_Agree_) is shown in Fig 5. Nodule subtlety, margin, texture, lobulation, malignancy, and nodule volume ($\bar{V_{manual}}$) were positively and significantly correlated to the DSI_Agree_ (p_t-test_ range: 1.5x10^-9^ - 6x10^-3^) (Fig 5). As the nodule volume increased from 162ml to 796ml, the median DSI_Agree_ increased from 55% to 78%. The median agreement between CIP and manual segmentations increased from 56% to 70% as the likelihood of the nodule malignancy increased.

An example of a non-solid subtle nodule with poorly defined boundaries is shown in Fig 4D. The accuracy of the CIP segmentation was poor for non-solid or semi-solid nodules, or nodules with poorly defined boundaries and subtle appearances with a median DSI_Agree_ ranging from 15%–41% (Table 2). The performance of CIP segmentations for solid nodules with sharp margins and obvious appearances increased to 61% (Table 2). Nodules that were not marked to be lobulated or spiculated by the radiologists had a median DSI_Agree_ of 59%. Substantial agreement (median DSI_Agree_ > 65%) between CIP and manual segmentations were found in nodules with marked lobulation and spiculation (Table 2). Nodule sphericity (p_t-test_ = 0.94) and calcification (p_Kruskal-Wallis_ = 0.49) were not significantly correlated with $\bar{V_{manual}}$. Median DSI_Agree_ was ~60% for all nodules regardless of the nodule sphercitiy and calcification conditions (Fig 5, Table 2).

**Table 2:** Distribution of nodule characteristics. Median $\bar{V_{manual}}$, median DSI_Agree_ and their corresponding interquartile ranges (IQR) for each nodule characteristic.

Nodule characteristics	Scoring	Total (n = 354 nodules) Number (%)	Median DSI_Agree_ (IQR)	Median $\bar{V_{manual}}$ (IQR)
Subtlety	1 = Extremely subtle	5 (1.0%)	19% (5–24%)	207ml (109–380ml)
	2 = Moderately subtle	7 (2.0%)	61% (49–62%)	108ml (97–130ml)
	3 = Fairly subtle	69 (20%)	56% (34–62%)	172ml (106–273ml)
	4 = Moderately subtle	87 (25%)	52% (42–67%)	209ml (148–414ml)
	5 = Obvious	186 (53%)	64% (53–79%)	602ml (289–1415ml)
Calcification	1 = Popcorn	0 (0%)	N/A	N/A
	2 = Laminated	0 (0%)	N/A	N/A
	3 = Solid	57 (16%)	56% (47–62%)	220ml (124–374ml)
	4 = Non-central	0 (%)	N/A	N/A
	5 = Central	1 (0%)	59% (59–59%)	146ml (146–146ml)
	6 = Absence	296 (84%)	61% (45–74%)	370ml (172–884ml)
Sphercitiy (Roundness)	1 = Linear	0 (0%)	N/A	N/A
	2	3 (1.0%)	63% (61–66%)	497ml (403–516ml)
	3 = Ovoid	90 (25%)	60% (48–69%)	290ml (148–529ml)
	4	156 (44%)	60% (44–72%)	301ml (162–805ml)
	5 = Round	105 (30%)	58% (43–76%)	363ml (188–1001ml)
Margin	1 = Poorly	3 (1.0%)	6% (5–13%)	803ml (456–908ml)
	2	17 (5.0%)	50% (12–61%)	436ml (212–810ml)
	3	22 (6.0%)	51% (25–70%)	311ml (153–780ml)
	4	81 (23%)	59% (45–70%)	375ml (153–807ml)
	5 = Sharp	231 (65%)	61% (50–74%)	291ml (164–749ml)
Lobulation	1 = None	290 (82%)	59% (45–69%)	275ml (150–617ml)
	2	38 (11%)	62% (51–72%)	660ml (318–1930ml)
	3	15 (4.0%)	70% (57–83%)	809ml (609–2986ml)
	4	7 (2.0%)	80% (65–82%)	1357ml (815–1980ml)
	5 = Marked	4 (1.0%)	66% (39–84%)	1492ml (283–2801ml)
Spiculation	1 = None	312 (88%)	59% (45–70%)	291ml (154–693ml)
	2	28 (8.0%)	61% (54–72%)	483ml (207–785ml)
	3	7 (2.0%)	75% (70–81%)	1542ml (1285–4174ml)
	4	2 (1.0%)	83% (79–86%)	3064ml (2351–3777ml)
	5 = Marked	5 (1.0%)	81% (66–83%)	3036ml (984–7489ml)
Texture	1 = Non-Solid/GGO	13 (4.0%)	8% (3–21%)	380ml (212–810ml)
	2	2 (1.0%)	48% (46–49%)	200ml (149–251ml)
	3 = Part Solid/Mixed	7 (2.0%)	32% (17–58%)	711ml (315–815ml)
	4	4 (1.0%)	59% (53–65%)	357ml (264–440ml)
	5 = Solid	328 (93%)	61% (48–73%)	298ml (162–784ml)
Malignancy	1 = Highly unlikely	69 (20%)	56% (47–63%)	219ml (122–421ml)
	2 = Moderately unlikely	27 (8%)	56% (42–67%)	159ml (119–335ml)
	3 = Indeterminate	176 (50%)	58% (43–70%)	265ml (153–504ml)
	4 = Moderately suspicious	49 (14%)	67% (54–80%)	803ml (507–1073ml)
	5 = Highly suspicious	33 (9%)	73% (61–85%)	2573ml (1180–4489ml)

The intermediate values for Roundness, Margin, Lobulation, Spiculation, and Texture are allowed to use by the radiologists. N/A = not applicable. GGO = Ground Glass Opacity

While the interior structure of all the other 343 nodules was scored as soft tissue, one nodule was rated to be air (Fig 4C). For this nodule, the CIP segmentation failed to identify the boundary of the nodule resulting in a DSI_Agree._ of 1% and was unstable (dsi_CIP_ = 42%) (Fig 4C). Nodule malignancy, subtlety, calcification, lobulation, and spiculation were positively and significantly correlated to $\bar{V_{manual}}$ (p_t-test_ range = 6.87x10^-27^–1.12x10^-4^).

As image voxel thickness increased from 1 mm to 5 mm, the median DSI_Agree_ increased from 62% to 79%. However, of the 354 CT images, only two images had a thickness of 5 mm. After excluding these two images from the analysis, the influence of image thickness on the accuracy of CIP segmentation was insignificant and was nearly negligible (Spearman’s correlation coefficient of 0.01 and p_t-test_ of 0.82).

According to visual inspection, 13% (47/354) of the nodules did not require any manual adjustment. Minor to moderate manual adjustments were needed for 37% (129/354) of nodules that included non-nodular tissues (e.g. pleura). Substantial manual adjustment were required for 50% (176/354) of the nodules.

## Discussion

Pulmonary nodules can indicate early stage lung cancer or a number of benign conditions. CT-based imaging features have been used to generate imaging biomarkers that predict the malignancy of lung nodules and have demonstrated promising results [19, 20]. Careful delineation of the lung nodule volumes is required for accurate feature extraction to build these imaging biomarkers [15–18]. Most commonly, manual segmentation is the method of choice; however, manual segmentation is not only time consuming, but is also affected by inter-observer variability [21, 22, 24]. Although many automatic and semi-automatic segmentation algorithms for nodule segmentation have been proposed, the widespread use of these algorithms, in the scientific and clinical communities, is hampered by their limited accessibility. In this study, we compared the robustness of manual segmentation and a publically accessible nodule segmentation algorithm, known as CIP segmentation.

CIP segmentation may potentially provide a reliable way to assist physicians in the nodule delineation process by reducing inter-observer variability and the physician workload. The CIP segmentations computed from different seed points from the four radiologists were in excellent agreement, indicating that the CIP method is robust and stable to different segmentation seed points. In comparison, manual segmentation was significantly less stable than CIP segmentation. Comparatively, Velazquez et al (2013) assessed the robustness of manual delineations and a 3D Slicer semi-automatic algorithm, known as GrowCut, in defining the volume of twenty non-small cell lung (NSCLC) tumors [30]. They found that the GrowCut algorithm resulted in significantly smaller regions of uncertainty than manual delineations and concluded that it could be used as a starting point for tumor target delineation in radiotherapy and high-throughput data mining research when manual delineations are not available. The results of our study are consistent with their findings that semiautomatic algorithms (in our case, CIP segmentations) are more stable than manual segmentations in defining lung nodule volumes. Furthermore, CIP segmentation is efficient with a median computation time of only 10s on a personal computer. We anticipate that the computational time of the CIP segmentation algorithm would be significantly reduce on a more powerful computer.

Despite the potential applications of the CIP segmentation algorithm, manual adjustment of the segmentations may be needed, especially for small nodules and nodules with poorly defined boundaries, subtle appearance, and non-solid or part-solid textures. Nodule calcification and sphericity have no impact on the performance of CIP segmentations. The accuracy of CIP segmentations tended to be better when the nodule was solid, more obvious, and with a sharp boundary. Non- and part-solid nodules with a hazy appearance failed to completely obscure parenchymal structures, and have been therefore difficult detect and segment by many segmentation algorithms [27, 28, 38, 39]. Similarly, CIP segmentations also suffer from this limitation, where nodules with subtle appearances may have similar image density as its background that makes the full extent of nodules difficult to define. Therefore, in these cases, the knowledge of experienced radiologists is needed to estimate the extent (or boundary) of the nodules and manually edited the CIP segmentation. The robustness of the CIP segmentation was nearly perfect and significantly better than the manual segmentations (Fig 2). Hence, the robustness of manual adjustment based on the CIP segmentation is anticipated to be superior to manual segmentation, but not as robust as CIP segmentation alone.

Several segmentation algorithms have been proposed to improve the contours of structures with hazy appearances, such as non- and part-solid nodules, such as the Markov random field theory-based algorithm [40, 41], neural network [42], and a hybrid algorithm that combines threshold-based region growing, connected component analyses and convex hull calculations [28, 39, 43]. Brief descriptions of five example algorithms that have good performance in segmenting GGO and partly solid nodules are shown in Table 3. However, these more sophisticated algorithms are not easily accessible and have not been implemented into open source platforms for widespread use. Incorporating algorithms for defining non- and sub-solid nodules into the 3D Slicer CIP can further improve the performance of the CIP segmentations. Furthermore, although juxtapleural nodules and nodules with vessel attachment are more challenging to segment than the isolated nodules [44, 45], these nodules characteristics were not evaluated and scored by the LIDC radiologists. In the future, it would be interesting to compare the performance of the CIP segmentation in delineating juxtapleural, vessel-attached, and isolated nodules.

**Table 3:** Brief descriptions of five algorithms for ground glass opacity (GGO) or partly solid nodule segmentation.

Authors	Description	Number of manually segmented nodules for validation
Lassen et al (2015) [28]	Regions of nodules and parenchyma were initially segmented based on the threshold-based region growing algorithm, followed by chest wall removal using the connected component analysis and convex hull calculation. Attached vessels are removed by morphological operations.	59 LIDC lung nodules
Tan et al (2012) [41]	Marker-controlled watershed were geometric active contours with Markov random field for segmenting nodules	23 LIDC lung and 22 phantom nodules
Zhu et al (2011) [40]	Image intensity distributions of the lung nodules were modeled using simple and adaptive data. Gibbs sampler was used to solve the maximum a posteriori probability estimator to identify the best nodule segmentation.	41 lung nodules acquired at the Huadong Hospital, Shanghai, China
Kubota et al (2010) [38]	Lung parenchyma and nodule-like structures (foreground) were separated using couple competition and diffusion processes. The Euclidean distance transformation was then performed to identify the core of a nodule. Attached structures were removed by a region growing algorithm on the Euclidean distance map. Finally, the segmentation was defined by the overlap region of the nodule’s convex hull and the foreground.	105 LIDC nodules
Zhou et al (2006) [39]	GGO nodules were first detected using the boosted k-nearest neighbor. The nodules were then segmented based on the nonparametric density estimation of a 3D texture probability map. Eigenvalues analysis of Hessian matrix was used to remove tube like vessel structures.	10 clinical nodules

CIP segmentations may overestimate nodule region of interest for small nodules. A previous study used eighteen nodules of different sizes, shapes and densities that were embedded into various locations of an anthropomorphic thorax phantom to validate the CIP segmentation algorithm [33]. On average, CIP segmentation overestimated the phantom nodule volume by 35%. In our study, CIP segmentation performed best for large nodules with a difference between segmented and phantom nodule volumes <15%. In patients, larger differences were found between the CIP and manually segmented nodule volumes ($\bar{V_{CIP}}$ = 477ml vs $\bar{V_{manual}}$ = 309ml). This may due to the fact that patient nodules (e.g. vessel-attached and juxtapleural nodules) were more variable than those embedded in the phantom. CIP segmentations performed better for nodules with larger volumes ($\bar{V_{manual}}$) and a higher likelihood of being malignant. Nodules that are larger in size (e.g. >4mm nodule diameter in the National Lung Screening Trail in the United Stated [5] are generally considered to be more likely to be malignant. Moreover, the appearance of a large nodule is less subtle and more obvious. As expected, in our study, nodule malignancy and subtlety were positively correlated with nodule volumes. According to the LIDC publications and documentations, it is unclear whether all segmentations were performed by the same or different radiologists [23, 42, 46]. However, we found that the nodule volumes segmented by different LIDC radiologists were consistent and in excellent agreement (Fig 1). Thus, our comparison between CIP- and manual segmentations would be mildly influenced by the inter-radiologist variability even if the nodules were not defined by the same radiologist. Substantial agreement between CIP and manual segmentations were found for nodule volumes >796ml. Moreover, larger nodule volumes may be more likely to be lobulated and spiculated due to the significant correlation between these characteristics and the nodule volume. This may explain why the CIP segmentation method performed better for nodules with marked lobulation and spiculation. We observed that nodule volumes computed from CIP segmentations were significantly greater than those computed from manual segmentation. For nodules with smaller size, CIP segmentations often include adjacent tissues, such as normal lung and blood vessels. Furthermore, small nodules were not only more likely to have subtle appearances and thus, were difficult to detect, but could also be easily overestimated by CIP segmentations. Therefore, manual adjustments may be needed to correct for the overestimation of the small nodules in the CIP segmentations.

An emerging field that converts medical images into high dimensional mineable data is called radiomics [47]. In addition to differentiating between benign and malignant nodules, radiomic features of lung lesions could also be used to predict clinical outcomes and treatment response [1, 2, 48]. Several lung screening trials using CT images have been launched in Asia [49–51], Europe [52–54], and the United States [5, 55, 56] to identify patients with early lung cancer. Due to the easy accessibility of the CIP segmentation algorithm, this method may be useful for nodule delineation in these lung trial datasets that consist of a large number of patients. This could subsequently expedite the high-throughput extraction of imaging features for radiomic analysis for nodule classification and patient outcomes for precision medicine, especially for patients with large nodules. Future studies will need to investigate how inaccurate CIP segmented nodule volumes influence the predictive power of radiomic features.

The CIP segmentation algorithm relies on several parameters for the generation of the feature maps that were experimentally set up to default values. Further improvements in the segmentation result may be expected by a careful selection of those parameters. Our experience was that the default parameters provided in CIP work well on average but specific nodule characteristics would benefit for tailored parameters selection.

## Conclusion

A semi-automatic segmentation algorithm implemented under the 3D Slicer Chest Imaging Platform (CIP) may be useful for assisting physicians in nodule volume delineation. CIP segmentations can potentially reduce the physician workload in 13% of the nodules and inter-observer variability due to its computational efficiency and superior stability compared to manual segmentation. Due to the public accessibility of the CIP segmentation algorithm, it can be employed to initiate nodule segmentation for large datasets, such as lung screening trials, thereby facilitating efficient nodule classification and high-throughput data mining research. However, CIP segmentations should be used with care and manual adjustment of the segmentations may be needed for the majority (87%) of the nodules, including small nodules, and nodules with subtle appearances, poorly defined boundaries and non- and part-solid texture. Although manual adjustment is needed for many cases, CIP segmentation provides a preliminary contour for physicians as a starting point

## Supporting information

S1 File Examples of the segmentation artifact and the distribution of radiologist rating for each nodule characteristics. (DOCX)
