# Supplementary material for: Application of the 3D slicer chest imaging platform segmentation algorithm for large lung nodule delineation
Source: PLoS One. 2017 Jun 8;12(6):e0178944. doi: 10.1371/journal.pone.0178944 (PMC5464594; doi:10.1371/journal.pone.0178944)
Supplement: S1 File — (DOCX) [file pone.0178944.s001.docx]

S1 File. Examples of the segmentation artifact and the distribution of radiologist rating for each nodule characteristics.


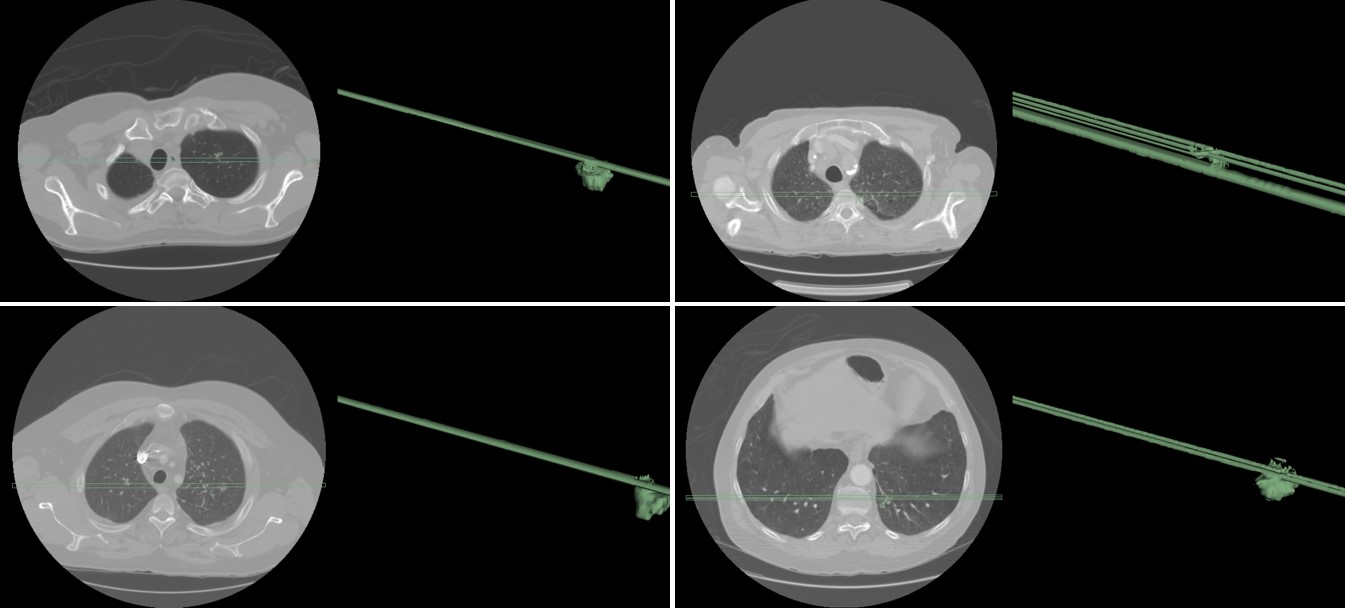


Fig A. Examples of the segmentation artifact. 60 patients (77 nodules) with imaging artifacts in segmentations were excluded from the study.


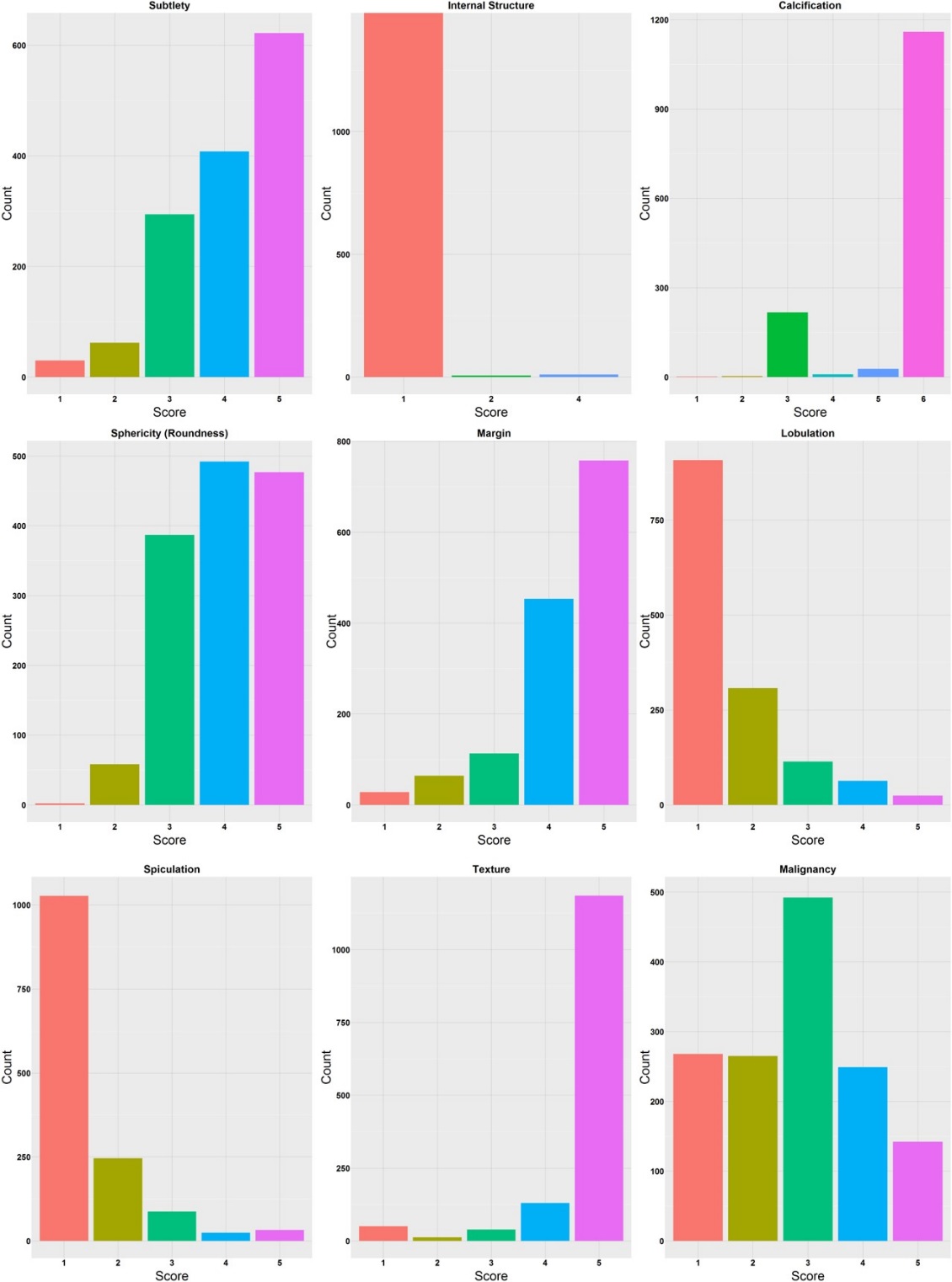


Fig B. Distribution of radiologist rating for various nodule characteristics.
